# Raised sputum extracellular DNA confers lung function impairment and poor symptom control in an exacerbation-susceptible phenotype of neutrophilic asthma

**DOI:** 10.1186/s12931-021-01759-z

**Published:** 2021-06-03

**Authors:** Mustafa Abdo, Mohib Uddin, Torsten Goldmann, Sebastian Marwitz, Thomas Bahmer, Olaf Holz, Anne-Marie Kirsten, Frederik Trinkmann, Erika von Mutius, Matthias Kopp, Gesine Hansen, Klaus F. Rabe, Henrik Watz, Frauke Pedersen, the ALLIANCE study group

**Affiliations:** 1 LungenClinic Grosshansdorf, Airway Research Center North (ARCN), German Center for Lung Research (DZL), Wöhrendamm 80, 22927 Grosshansdorf, Germany; 2 Respiratory and Immunology, BioPharmaceuticals R&D, AstraZeneca, Gothenburg, Sweden; 3 Research Center Borstel, Airway Research Center North (ARCN), German Center for Lung Research (DZL), Borstel, Germany; 4 Department for Internal Medicine I, Airway Research Center North (ARCN), German Center for Lung Research (DZL), University Hospital Schleswig-Holstein-Campus Kiel, Kiel, Germany; 5 Fraunhofer ITEM, Biomedical Research in Endstage and Obstructive Lung Disease Hannover (BREATH), German Center for Lung Research, Hannover, Germany; 6 Pulmonary Research Institute at the LungenClinic Grosshansdorf, Airway Research Center North (ARCN), German Center for Lung Research (DZL), Grosshansdorf, Germany; 7 Department of Pneumology and Critical Care Medicine, Thoraxklinik, University of Heidelberg, Translational Lung Research Center Heidelberg (TLRC), German Center for Lung Research (DZL), Heidelberg, Germany; 8 Department of Biomedical Informatics, Heinrich-Lanz-Center, University Medical Center Mannheim, Mannheim, Germany; 9 Dr Von Hauner Children’s Hospital, Ludwig Maximilians University of Munich, Comprehensive Pneumology Center Munich (CPC-M), German Center for Lung Research (DZL), Munich, Germany; 10 Department of Pediatric Pneumology, Inselspital, University Children’s Hospital of Bern, University of Bern, Bern, Switzerland; 11 Division of Pediatric Pneumology and Allergology, University Hospital Schleswig-Holstein-Campus Luebeck, Airway Research Center North (ARCN), German Center for Lung Research (DZL), Luebeck, Germany; 12 Department of Paediatric Pneumology, Allergology and Neonatology, Hannover Medical School, Biomedical Research in Endstage and Obstructive Lung Disease (BREATH), German Center for Lung Research (DZL), Hannover, Germany

**Keywords:** Extracellular DNA, Neutrophil extracellular traps, Neutrophilic asthma, Asthma outcomes

## Abstract

**Background:**

Extracellular DNA (e-DNA) and neutrophil extracellular traps (NETs) are linked to asthmatics airway inflammation. However, data demonstrating the characterization of airway inflammation associated with excessive e-DNA production and its impact on asthma outcomes are limited.

**Objective:**

To characterize the airway inflammation associated with excessive e-DNA production and its association with asthma control, severe exacerbations and pulmonary function, particularly, air trapping and small airway dysfunction.

**Methods:**

We measured e-DNA concentrations in induced sputum from 134 asthma patients and 28 healthy controls. We studied the correlation of e-DNA concentrations with sputum neutrophils, eosinophils and macrophages and the fractional exhaled nitric oxide (FeNO). Lung function was evaluated using spirometry, body plethysmography, impulse oscillometry and inert gas multiple breath washout. We stratified patients with asthma into low-DNA and high-DNA to compare lung function impairments and asthma outcomes.

**Results:**

Patients with severe asthma had higher e-DNA concentration (54.2 ± 42.4 ng/µl) than patients with mild-moderate asthma (41.0 ± 44.1 ng/µl) or healthy controls (26.1 ± 16.5 ng/µl), (all p values < 0.05). E-DNA concentrations correlated directly with sputum neutrophils (R = 0.49, p < 0.0001) and negatively with sputum macrophages (R = − 0.36, p < 0.0001), but neither with sputum eosinophils (R = 0.10, p = 0.26), nor with FeNO (R = − 0.10, p = 0.22). We found that 29% of asthma patients (n = 39) had high e-DNA concentrations above the upper 95th percentile value in healthy controls (55.6 ng /μl). High-DNA was associated with broad lung function impairments including: airflow obstruction of the large (FEV_1_) and small airways (FEF50%, FEF25–75), increased air trapping (RV, RV/TLC), increased small airway resistance (R5-20, sReff), decreased lung elasticity (X5Hz) and increased ventilation heterogeneity (LCI), (all P values < 0.05). We also found that high e-DNA was associated with nearly three-fold greater risk of severe exacerbations (OR 2·93 [95% CI 1.2–7.5]; p = 0·012), worse asthma control test (p = 0.03), worse asthma control questionnaire scores (p = 0.01) and higher doses of inhaled corticosteroids (p = 0.026).

**Conclusion:**

Increased production of extracellular DNA in the airway characterizes a subset of neutrophilic asthma patients who have broad lung function impairments, poor symptom control and increased risk of severe exacerbations.

## Background

Asthma is a heterogeneous disease that comprises several clinical phenotypes [1]. A considerable proportion of asthma patients are thought to have the neutrophilic phenotype [2] which is characterized by high sputum neutrophil counts and is linked to asthma severity [3], frequent exacerbations [4], and steroid resistance [5]. Unravelling the role of neutrophils in the pathophysiology of asthma is still ongoing and has the potential to reveal novel molecular mechanisms and druggable targets. Recent studies have begun to reveal the potential pro-inflammatory role of extracellular DNA (e-DNA) released by activated airway neutrophils in asthma [6, 7]. Extracellular DNA forms web-like scaffolds in combination with histones and neutrophil granular proteins, such as myeloperoxidase (MPO) and neutrophil elastase (NE), called the neutrophil extracellular traps (NETs) [8]. Although NETs are important components of the antimicrobial innate immunity, aberrant NETs production might be harmful to the airway tissue through the excessive release of the histotoxic components of NETs, including proteases (MPO, NE) into the extracellular surroundings. This can promote airway mucosal inflammation, induce epithelial cell death and contribute to airway mucus hypersecretion, causing NETopathic airway inflammation [9, 10]. Recent work by Lachowicz-Scroggins and colleagues showed that high sputum e-DNA concentration in asthma patients was accompanied by raised sputum neutrophils and was associated with activation of innate immune responses and elevated sputum cytokines (e.g. IL-1β) [6].

Taken together, excessive e-DNA formation and the subsequent NETopathic inflammation point to a potential pathobiological role in asthmatic inflammation, but insights into broader asthma outcomes are limited. In particular, there is a lack of data demonstrating the interplay between high e-DNA with asthma control and pulmonary function, particularly, lung function measures beyond airflow obstruction, such as air trapping and small airway dysfunction. Furthermore, there are still uncertainties about the characterization of airway inflammation associated with high e-DNA production in asthma, as airway eosinophils might also contribute to e-DNA production [11].

Therefore, we evaluated the association of sputum e-DNA concentration with differential sputum cells concentrations, pulmonary function, symptom control and the frequency of severe exacerbations in asthma patients.

## Methods

### Study design

Eligible subjects were adults with asthma and healthy controls who were recruited to the multicenter prospective longitudinal All Age Asthma Cohort (ALLIANCE), a cohort of pediatric and adult patients with asthma in Germany, initiated by the German Centre for Lung Research (DZL). The study was approved by a local Ethics Committee at the Medical School of Lübeck (Az.21-215) and is registered at clinicaltrials.gov (adult arm: NCT02419274). All subjects provided written informed consent prior to enrollment. Detailed information regarding recruitment, inclusion and exclusion criteria was described previously [12]. This study was a cross-sectional analysis on the baseline data. Sputum specimens were available for 142 asthma patients and 34 healthy controls. Six healthy controls and eight asthma patients were excluded due to insufficient sputum quality. Eventually, 162 subjects were included in our data analysis. The subjects had to have stable disease without acute exacerbations or respiratory tract infections within four weeks prior to study visit. Asthma patients with current or former smoking history were also included. Healthy controls were never-smokers, had normal spirometry and no history of lung diseases.

### Sputum preparation and extracellular DNA quantification

Sputum was induced and processed according to standardized procedures [13]. Briefly, patients inhaled hypertonic nebulized saline (3–4–5%, each 7 min) for a total inhalation time of 21 min. Induction was discontinued if FEV1 fell by more than 20% as compared with baseline. Sputum plugs were selected within one hour after induction. The sputum plugs of all inhalation periods were pooled, weighted and incubated with four volumes of 0.1% dithiothreitol (DTT, Sputolysin®; Calbioch, Bad Soden, Germany) for 15 min at room temperature. Thereafter, 2 volumes of phosphate buffered saline (PBS) were added and cell suspension was filtered through a 70 µm cell strainer. Subsequently, cell suspension was centrifuged (600 g, 4 °C, 10 min). Supernatants for e-DNA quantification were frozen at − 80 °C. The remaining cell pellets were resuspended in PBS. Total cell counts and viability were determined by haemacytometer and trypan blue (Sigma, Deisenhofen, Germany) staining. Cytospins were prepared and differential cell counts analyzed as previously described [13]. Sputum supernatant samples for extracellular-DNA quantification were thawed and mixed thoroughly. 1 µl of supernatant was transferred to the measurement pedestal of the NanoDrop 2000 spectrophotometer (Thermo Fischer Scientific, Inc., Waltham, MA). Extracellular DNA concentration was measured by determining the absorbance at 260 nm. All measurements were performed in duplicate according to manufacturer’s manual (https://assets.thermofisher.com/TFS-Assets/CAD/manuals/NanoDrop-2000-User-Manual-EN.pdf). A mixture of four volumes of 0.1% DTT and 3 parts PBS was used as negative control [14].

We classified patients with asthma into high-DNA and low-DNA to study the association of sputum e-DNA with sputum cell concentrations, pulmonary function and asthma outcomes. Further, we compared e-DNA concentrations between patients based on their sputum inflammatory cellular patterns i.e. (eosinophils ≥ 2%, eosinophils ≥ 3%, neutrophils ≥ 40% and neutrophils ≥ 65%) [2, 15].

### Asthma severity and symptom control

We defined severe asthma based on European Respiratory Society/American Thoracic Society guidelines [16]. Further, asthma control was assessed by the asthma control test (ACT), asthma control questionnaire (ACQ5) and the annualized number of severe exacerbations 12 months prior to study visit, defined as a burst of systemic corticosteroids for ≥ 3 days [16].

### Lung physiology characteristics

We performed spirometry, body plethysmography, impulse oscillometry (IOS) and inert gas multiple breath washout (MBW) in accordance to current ERS recommendations [17–20]. Measures of airflow obstruction were the forced expiratory volume in the first second (FEV_1_) and its ratio to the forced vital capacity (FVC). Measures and indirect markers of small airway function were: mean forced expiratory flow at 50% and between 25 and 75% of the forced vital capacity (FEF50%, FEF25–75%) from forced spirometry, specific effective airway resistance (sReff), residual lung volume (RV), and ratio of RV to total lung capacity (RV/TLC) from body plethysmography, the small airway resistance (R5Hz-20 Hz) and lung reactance at 5 Hz (XHz) from IOS, and lung clearance index (LCI) measured by MBW.

### Statistical analysis

We used the unpaired *t*-test, Wilcoxon rank test or Fisher exact test to determine the significance of differences in clinical variables between the study groups. We used Fisher's exact test to determine the odds ratio of severe exacerbation in the study groups. Spearman's rank correlation was used to test for statistical dependence between two variables. Statistical analyses were performed using R (version 3.6.2; R Foundation, Vienna, Austria). An alpha error of less than 5% was considered statistically significant.

## Results

**Fig. 1:**
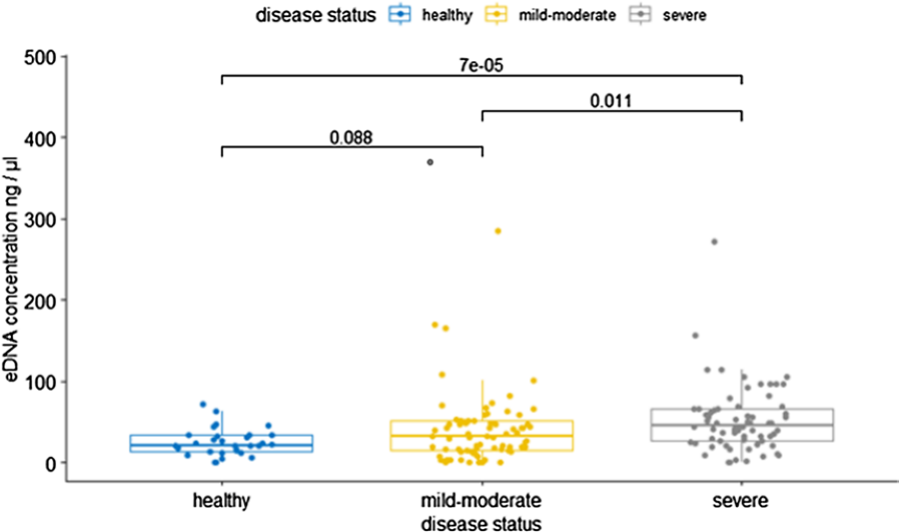
Sputum e-DNA concentrations in asthma patients and healthy controls: the concentrations were (26.1 ± 16.5, 41.0 ± 44.1 and 54.2 ± 42.4 ng/µl) in healthy controls, mild-moderate asthma and severe asthma, respectively. P-values are from Wilcoxon–Mann–Whitney-Test

We included 70 patients with mild to moderate asthma, 64 patients with severe asthma and 28 healthy controls. Compared to healthy controls, patients with asthma were older, heavier and had increased neutrophil and eosinophil concentrations in blood and sputum. Detailed clinical characteristics are given in Table 1. Furthermore, asthma patients had significantly increased sputum e-DNA concentrations compared to healthy subjects (47.3 ± 43.6 vs 26.1 ± 16.5 ng/µl, p = 0.002). The concentration of sputum e-DNA was also elevated in severe asthma compared to mild-moderate asthma patients (Fig. 1).

**Table 1:** Characteristics of healthy subjects and patients with asthma

Characteristic	Healthy (*n* = *28*)	Asthma (*n* = *134*)	P-value
Age, years	41.4 ± 18	52.3 ± 12	0.002
Sex, % male	53	47	0.54
BMI, kg/m^2^	25.2 ± 3.8	27.6 ± 5.1	0.016
Smoking history %	0	23	0.005
Current smokers, %	0	9	< 0.001
Severe asthma, %	–	48	–
FEV1, %	102	83.5	< 0.001
FVC, %	110	105	0.67
FEV1/FVC, %	77.5	65.1	< 0.001
Blood cell counts, 10^3^/µl			
Eosinophils	145 (90–182)	240 (140–450)	< 0.001
Neutrophils	2770 (2435–3452)	4210 (3370–5960)	< 0.001
Sputum cell counts, %*			
Eosinophils	0.1 (0.0–0.30)	1.5 (0.4–7.0)	< 0.001
Neutrophils	25.5 (14–51)	53.3 (32–71)	0.008
Macrophages	67.2 (44–81)	31 (15–53)	0.003

Data are reported in mean ± SD or median (interquartile range)

*BMI* body mass index, *FEV1* predicted forced expiratory volume in the 1 s, *FVC* forced vital capacity. Smoking history: current or former smokers with smoking history ≥ 10 pack years

*Sputum cell counts percentage of non-squamous cells

In healthy controls, we identified a cutoff value (55.6 ng/µl) at the 95th percentile to be the upper limit of normal for sputum e-DNA concentration. Accordingly, 29% of asthma patients (*n* = 39) had e-DNA concentrations more than 55.6 ng/ μl and were stratified as high-eDNA, remaining patients (*n* = 95) were stratified as low-eDNA. Mean e-DNA concentrations were 94.7 ± 52 and 27.8 ± 16 ng/μl in high and low-eDNA patients, respectively. Clinical features of high-eDNA and low-eDNA patients are represented in Table 2. Comparing high-DNA to low-DNA patients, we observed no statistically significant differences between both groups regarding demographics features, BMI or smoking habits (Table 2). A greater proportion of high-eDNA patients were found to have regular oral corticosteroids (OCS) with higher doses of both OCS and inhaled corticosteroids (ICS). However, only the difference in ICS dose was statistically significant (p = 0.026).

**Table 2:** Characteristics of patients with asthma classified by DNA concentration

Characteristic	Low-eDNA (*n = 95*)	High-eDNA (*n = 39*)	P-value
Age, years	52 (44–63)	51 (43–59)	0.57
Sex, % male	49	41	0.44
BMI, kg/m^2^	27.3 (24.0–29.0)	26.3 (23.8–34.4)	0.58
Smoking history %	20	27	0.37
Current smokers, %	9	10	1.0
Severe asthma, %	41	64	0.021
Severe exacerbations in last 12 months, %	49	74	0.012
ICS dose, µg	450 (250–950)	500 (450–1000)	0.026
OCS use, %	20	33	0.12
OCS dose; mg	7.5 (5–11)	12 (10–15)	0.14
Blood cell counts, 10^3^/µl			
Eosinophils	245 (140–477)	190 (135–450)	0.56
Neutrophils	4035 (3302–5758)	4810 (3415–6135)	0.24
Sputum cell counts			
Eosinophils, 10^6^/ml	0.03 (0.01–0.14)	0.06 (0.01–0.24)	0.29
Eosinophils, %*	1.5 (0.4–5.1)	2.0 (0.35–14.1)	0.87
Neutrophils, 10^6^/ml	0.81(0.31–1.83)	2.5 (1.0–5.8)	< 0.0001
Neutrophils, %*	48 (30–61)	65 (40–77)	0.005
Macrophages, 10^6^/ml	0.56(0.36–0.93)	0.67 (0.37–1.1)	0.43
Macrophages, %*	37 (18–56)	19 (11–34)	0.002
Sputum cell viability, %	80 (71–87)	80 (70–88)	0.93
Duration of sputum induction, min	21 (14–21)	21 (14–21)	0.30
FeNO, ppb	25 (15–46)	22 (11–35)	0.1
FEV1, %	87 (74–99)	76 (59–87)	0.002
FVC, %	109 (96–116)	104 (90–112)	0.06
FEV1/FVC, %	68 (61–73)	62 (54–72)	0.059
FEF50, %	58 (38–78)	37 (24–61)	0.004
FEF25–75, (l/s)	1.74 (1.2–2.3)	1.19 (0.64–2.0)	0.016
R20, KPa/l/s	0.32 (0.27–0.38)	0.33 (0.28–0.41)	0.29
R5, KPa/l/s	0.40 (0.35–0.50)	0.49 (0.38–0.62)	0.043
R5-20, %	32 (16–54)	51 (20–80)	0.058
X5Hz, KPa/l/s	− 0.12 (− 0.18–0.09)	− 0.20 (− 0.26–0.13)	< 0.001
RV, %	118 (103–132)	132 (107–164)	0.015
RV/TLC, %	36 (33–42)	41 (37–48)	0.012
sReff, %	98 (75–138)	133 (89–189)	0.016
LCI	6.1 (5.6–6.6)	6.9 (6.2–7.9)	< 0.001

Data are reported in median (interquartile range)

*BMI* body mass index, *ICS* inhaled corticosteroids, *ICS dose* fluticasone equivalent, *OCS* oral corticosteroids, *FeNO* fractional exhaled nitric oxide, *FEV1* forced expiratory volume in first second, *FVC* forced vital capacity, *FEF50% and FEF25–75* mean forced expiratory flow at 50% and between 25 and 75% of the forced vital capacity, *R20* proximal airway resistance at 20 Hz, *R5-20* small airway resistance (total lung resistance – large airway resistance), *X5* lung reactance at 5 Hz, *RV* residual volume, *TLC* total lung capacity, *sReff* specific effective airway resistance, *LCI* lung clearance index from multiple breath washout

*Sputum cell counts percentage of non-squamous cells

While patients with high-eDNA concentrations had markedly heightened sputum neutrophils (65%) compared to low-eDNA patients (48%), there were no significant differences in type 2 (T2) markers i.e. blood and sputum eosinophils and FeNO between both groups. However, interestingly, enumeration of sputum macrophage levels in patients with high-DNA showed significantly lower percentage of airway macrophages relative to low-DNA patients, (19% vs 37%; p = 0.002), respectively, suggesting a potential reciprocal relationship between sputum neutrophils and these phagocytes.

**Fig. 2:**
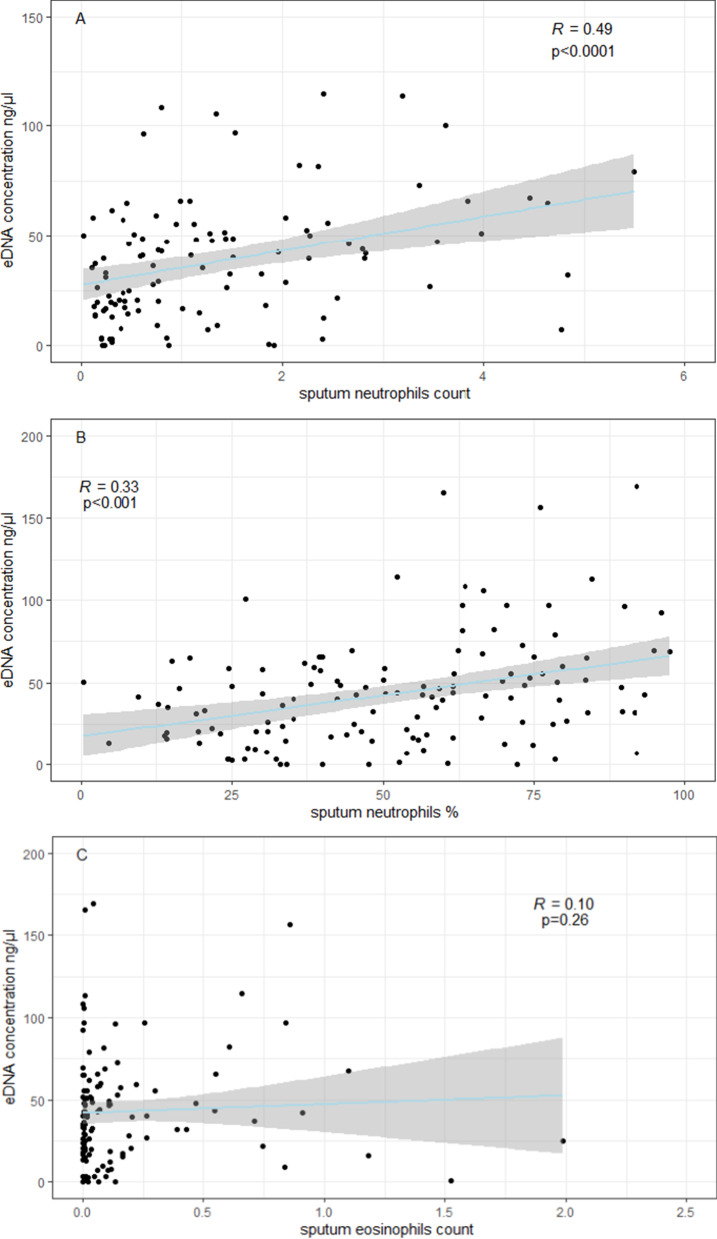

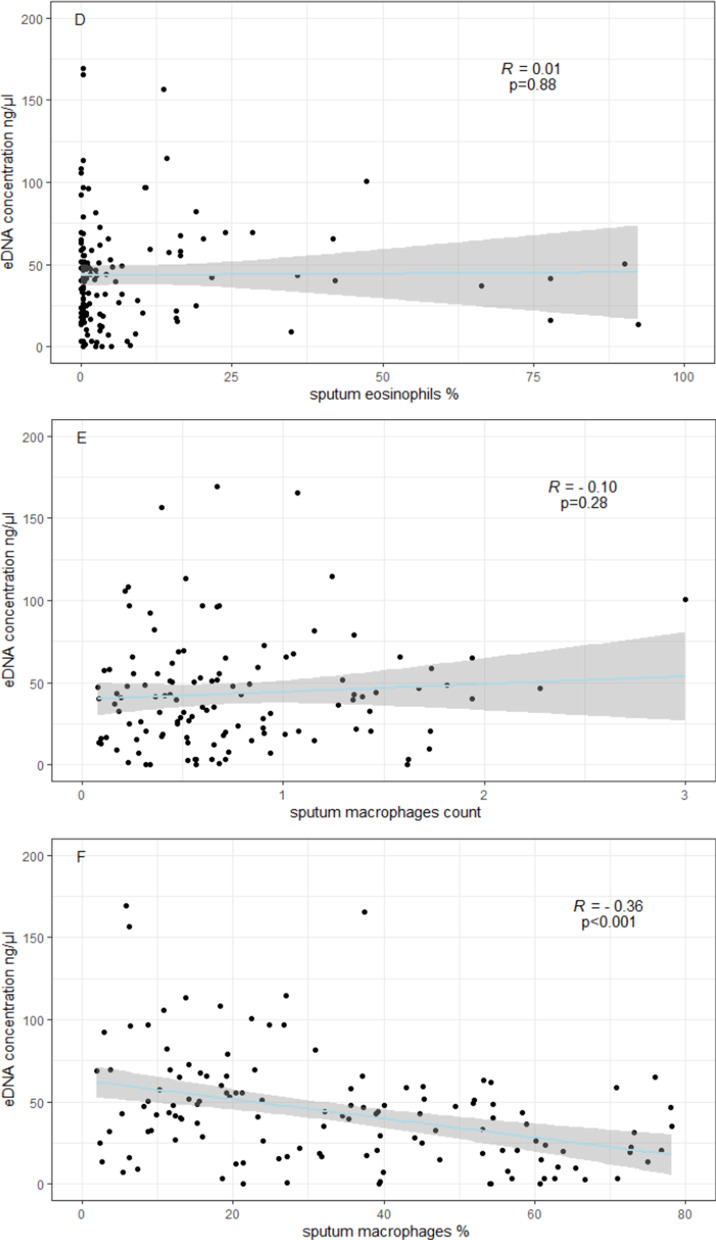

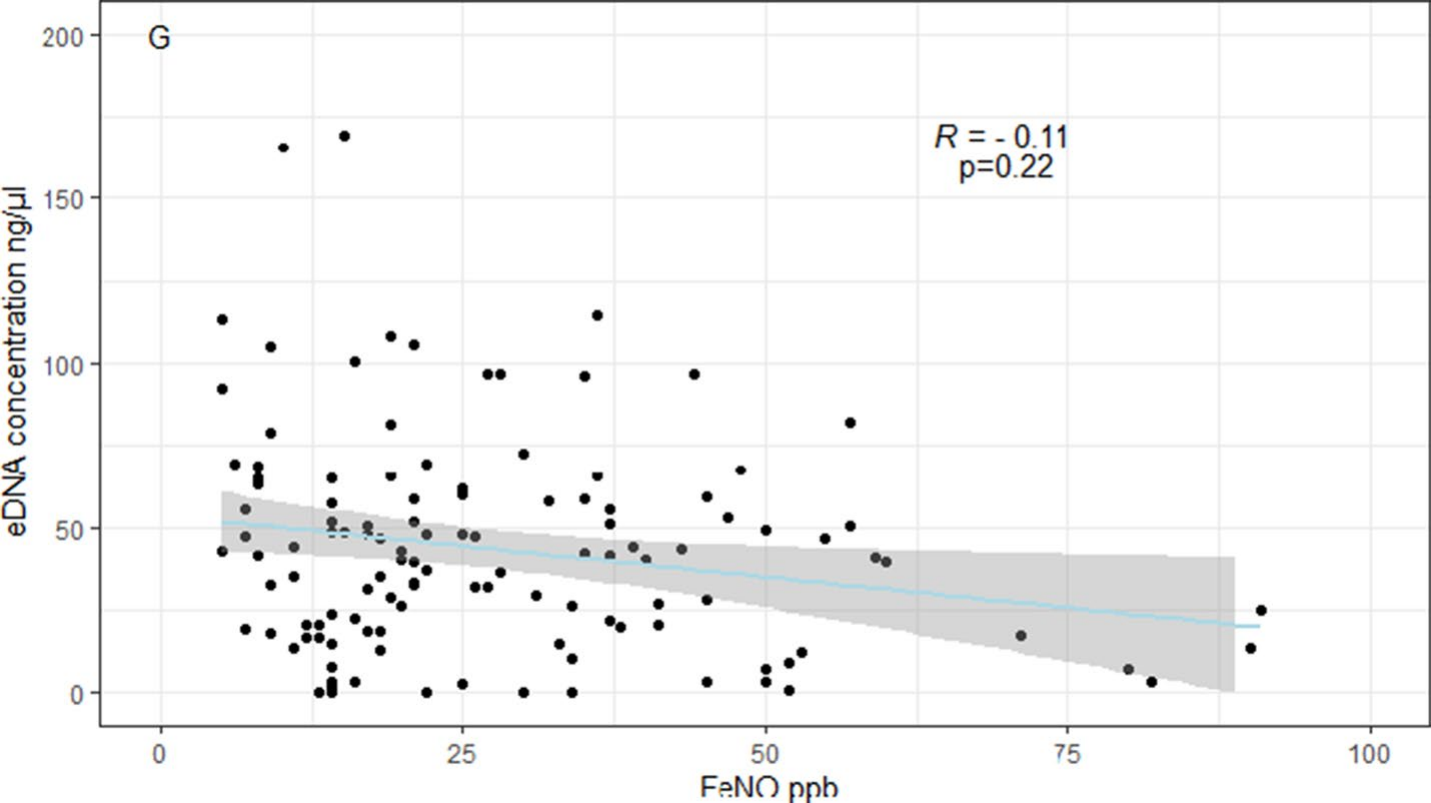
Spearman’s correlations between e-DNA concentrations and absolute sputum cell counts × 10^6^/ml (**A**, **C** and **E**), or sputum cell counts in percentage of sputum non-squamous cells (**B**, **D** and **F**) and with the fractional exhaled nitric oxide (FeNO) (parts per billion, ppb), (**G**)

Further, the mean concentration of sputum e-DNA in patients with eosinophilic asthma was similar to the e-DNA concentration in patients with non-eosinophilic asthma, both as defined by sputum eosinophils ≥ 2% (46.2 *vs* 41.3 ng/µl, p = 0.35) and sputum eosinophils ≥ 3% (43.6 *vs* 43.1 ng/µl, p = 0.97). By contrast, neutrophilic asthma patients had significantly higher e-DNA concentration compared to non-neutrophilic asthma patients, where neutrophilic asthma was defined as both sputum neutrophils ≥ 40%, (52.6 vs 36.3 ng/µl, p < 0.01) and sputum neutrophils ≥ 65% (63.28 vs 39.3 ng/µl, p < 0.001). Additionally, e-DNA concentrations correlated directly with sputum neutrophils counts (R = 0.49), p < 0.0001) and negatively with the percentage of sputum macrophages to sputum non-squamous cells (R = − 0.36, p < 0.0001), (Fig. 2a, f), but neither with sputum eosinophils nor with FeNO (Fig. 2c–e, g). The sputum analysis indicated good cell yield and viability in both groups (Table 2).

Furthermore, we noticed that high-DNA patients had worse lung function with airflow obstruction of the large (FEV_1_) and small airways (FEF50%, FEF_25–75_), in addition to increased air trapping (RV, RV/TLC), increased small airway resistance (R5-20, sReff), decreased lung elasticity (X5Hz), and increased ventilation heterogeneity (LCI) (Table 2). While the e-DNA concentration correlated similarly with the airflow obstruction in the large (FEV_1_) and small airways (FEF50%) as measured by spirometry (Table 3), it correlated even better with air trapping (RV/TLC) and ventilation heterogeneity (LCI).However, these correlations were fairly weak (Table 3).

**Table 3:** Univariate correlations between sputum e-DNA concentrations and lung physiology characteristics

Lung physiology characteristics	Spearman's correlation coefficient (ρ)	P value
FEV1, %	− 0.18	0.036
FVC, %	− 0.11	0.19
FEV1/FVC, %	− 0.14	0.11
FEF50, %	− 0.18	0.031
FEF25–75, (l/s)	− 0.16	0.06
R5-20, %	0.15	0.09
X5Hz, KPa/l/s	− 0.16	0.06
RV, %	0.24	0.004
RV/TLC, %	0.26	0.002
sReff, %	0.10	0.26
LCI	0.32	< 0.001

*FEV1* forced expiratory volume in first second, *FVC* forced vital capacity, *FEF50% and FEF25–75* mean forced expiratory flow at 50% and between 25 and 75% of the forced vital capacity, *R20* proximal airway resistance at 20 Hz, *R5-20* small airway resistance (total lung resistance – large airway resistance), *X5* lung reactance at 5 Hz, *RV* residual volume, *TLC* total lung capacity, *sReff* specific effective airway resistance, *LCI* lung clearance index from multiple breath washout

**Fig. 3:**
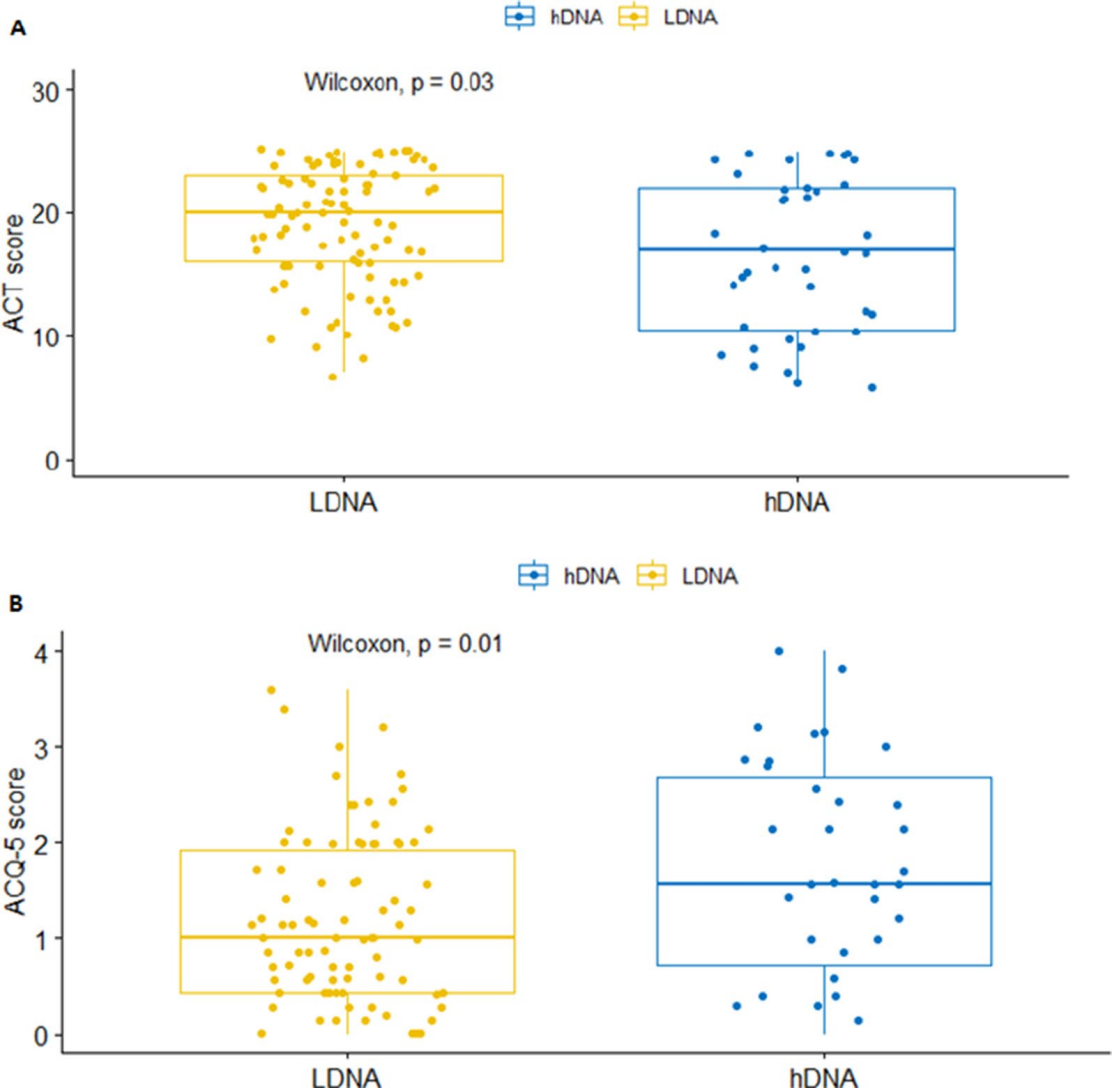
The association between sputum e-DNA concentration and symptom control: high-DNA is associated with poor symptom control. *ACT* asthma control test, *ACQ* asthma control questionnaire. P-values are from Wilcoxon–Mann–Whitney-Test

Furthermore, we also found that most of high-eDNA patients had severe asthma (Table 2) and poor symptom control (Fig. 3a, b). Of clinical relevance, the odds of severe asthma exacerbations were nearly three-fold greater in the high e-DNA asthma cohort than in the low e-DNA asthma cohort (OR 2.93 [95% CI 1.2–7.5]; p = 0.012) and these associations were suggestive of an exacerbation-susceptible phenotype of neutrophilic asthma.

## Discussion

Our findings indicate an upregulated extracellular DNA production in the sputum supernatants of asthma patients compared with healthy controls. They also indicate that the increase in sputum e-DNA production is associated with asthma severity. Furthermore, sputum e-DNA concentrations correlated directly with sputum neutrophil counts. By contrast, we found no significant correlations between sputum e-DNA concentrations and sputum eosinophil counts. Moreover, significantly lower levels of airway macrophages were noted in patients with high e-DNA accompanied by sputum neutrophilia compared to low-DNA patients. Heightened sputum e-DNA was associated with airflow obstruction in the proximal and distal airways, increased small airway resistance, air trapping and ventilation heterogeneity. Furthermore, heightened sputum e-DNA was associated with poor symptom control and increased risk of severe exacerbations, potentially indicating an exacerbation-susceptible phenotype of neutrophilic asthma.

We observed that 64% of the high-DNA patients had severe asthma versus 41% from patients with low-DNA (p = 0.021). In the study by Lachowicz-Scroggins and colleagues, they observed that heightened sputum e-DNA was associated with multiple clinical indicators of asthma severity, however, the distribution of severe asthma patients between low and high-eDNA patients was similar in their study (61% vs 66%) [6]. This discrepancy might be explained by the different cutoff value (3.8 μg/ml) that was used to define the upper limit of normal sputum e-DNA concentration which could also reflect the different e-DNA measuring techniques utilized in that study. The higher levels of sputum eDNA that we observed in more severe asthma patients are consistent with recent reports demonstrating that elevated levels of circulating NETs correlate with increasing asthma severity [7].

Furthermore, e-DNA concentrations correlated directly with sputum neutrophil counts but neither with sputum eosinophils nor with FeNO. This finding is consistent with previous findings that incriminated the activated airway neutrophils in excessive e-DNA production in asthma [6, 20]. Taken together, this implies that the major source of e-DNA in sputum is of neutrophil origin, even though also eosinophils might contribute to extracellular traps in airway mucosa of atopic asthmatics [11].

We also observed lower proportion of airway macrophages in patients with high e-DNA who had sputum neutrophilia compared to low-DNA patients. This finding is particularly interesting, because airway macrophages play a crucial role in the clearance of apoptotic neutrophils and cellular debris [21] to facilitate the resolution of inflammation. Previous studies have demonstrated defective neutrophil apoptosis and efferocytosis with an impaired phagocytic capacity of airway-derived macrophages in patients with neutrophilic airway inflammation including asthma [22–24]. It is reasonable to speculate whether a reduced number of airway macrophages observed could adversely impede phagocytic clearance to facilitate localized NET burden, potentially contributing to persistent NETopathic lung inflammation. However, a caveat with this premise is that we observed only significant lower proportion, but not absolute number, of sputum macrophages in patient with high-eDNA. Furthermore, the functional activity of airway macrophages may be more important than their cell numbers, thus, further studies are warranted to characterize the molecular mechanisms underlying the defects in macrophage functionality.

We observed that a numerically greater proportion of high-eDNA patients had regular OCS use with higher dosage. It was also noteworthy that the ICS dose was significantly higher in high-eDNA patients who also had a higher abundance of neutrophils sputum neutrophils. This might indicate that the presence of neutrophilic airway inflammation and the subsequent NET formation are not suppressible by corticosteroid treatment [25, 26], or even are attributable to increased airway neutrophils under systemic corticosteroids therapy [27]. Whether the sputum-enriched e-DNA is related to higher ICS doses or reflects a distinct pathobiology of severe neutrophil asthma is currently unclear; prospective mechanistic studies may provide further insights.

Moreover, upregulated sputum e-DNA production was associated with proximal airway obstruction and small airway disease. Accordingly, we also observed increased air trapping and ventilation heterogeneity in high-eDNA patients. Our findings regarding the correlation of sputum e-DNA with airflow obstruction are consistent with previous findings from patients with chronic obstructive pulmonary disease (COPD) [28] and cystic fibrosis (CF) [29]. It has been suggested that increased e-DNA production in the airways contributes to airflow obstruction by increasing the airway secretions [30] and mucus viscoelasticity [31], and by the contribution to airway inflammation through both activated proteases [32] and the proinflammatory cytokines [6]. Corresponding to lung function impairments, in our cohort, high-eDNA concentrations were also associated with nearly a threefold increase in the risk of severe exacerbations and frequent symptoms compared to low-eDNA patients with asthma, potentially indicating an exacerbation-susceptible phenotype of neutrophilic asthma.

Furthermore, we found that e-DNA concentrations in sputum were not affected by the current smoking status or former smoking history, in line with our previous finding where we showed that NET formation was not associated with current smoking in patients with COPD [14].

Our study has some limitations. First, we relied on extracellular DNA quantification rather than the semi-quantitative direct visualization of NETs. However, the quantification of extracellular DNA in sputum supernatants is representative and reliable method that has been validated as a surrogate marker for NETs in chronic lung diseases, including asthma [6, 14]. Second, we did not measure proteases and proinflammatory cytokines that trigger endogenous NET formation which might have supported our findings and helped understanding the mechanism of e-DNA induced airway inflammation.

In summary, we have established that the increased production of extracellular DNA in the airways characterizes a subset of neutrophilic asthma patients who have broad lung function impairments, poor symptom control with an exacerbation-susceptible phenotype. Mechanistically, we propose that both corticosteroid treatment and the impaired macrophage phagocytic capacity are potential mechanisms that might contribute to the presence of persistent neutrophilic airway inflammation and e-DNA/NET enriched microenvironment in asthma. Further studies are warranted to determine the precise mechanism of extracellular DNA in the pathophysiology of neutrophilic severe asthma.

## Data Availability

The datasets used during the current study are available from the corresponding author on reasonable request.

## Abbreviations

ACQ5: Asthma control questionnaire
ACT: Asthma control test
COPD: Chronic obstructive pulmonary disease
E-DNA: Extracellular DNA
FeNO: Fractional exhaled nitric oxide
ICS: Inhaled corticosteroid
OCS: Oral corticosteroid
NETs: Neutrophil extracellular traps
